# Treatment of Leucorrhœa, by Means of Tincture of Iodine

**Published:** 1844-10-19

**Authors:** 


### TREATMENT OF LEUCORRHŒA, BY MEANS OF TINCTURE OF IODINE.

M. Van Steenkiste has made use of a dilute tincture of iodine with great success in cases of obstinate chronic leucorrhœa.

℞. Iodine, gram. iv.; Alcohol, gram. lx. solve; et Aquæ distill. grammes cxxv.; about 30 fluid grammes (or f℥xv.) are to be thrown into the vagina as an injection, and repeated every day or every other day, according to the excitement it occasions.—*Ann. d'Obstetrique, cited in Gaz. des Hôpit.*

